# Biological screening of araripe basin medicinal plants using *Artemia salina* Leach and pathogenic bacteria

**DOI:** 10.4103/0973-1296.71792

**Published:** 2010

**Authors:** José Galberto M. da Costa, Adriana R. Campos, Samara A. Brito, Carla Karine B. Pereira, Erlânio O. Souza, Fabíola Fernandes G. Rodrigues

**Affiliations:** *Programa de Pós-Graduação em Bioprospecção Molecular, Departamento de Química Biológica, Laboratório de Pesquisa de Produtos Naturais, Universidade Regional do Cariri, Rua Cel. Antônio Luiz 1161, Pimenta, 63105-000 Crato-CE, Brasil*; 1 *Vice-Reitoria de Pesquisa e Pós-Graduação, Universidade de Fortaleza, Av. Washington Soares 1321, Edson Queiroz, 60811-905, Fortaleza-CE, Brasil*

**Keywords:** Araripe Basin, *Artemia salina*, ethanol extracts, larvicidal activities, pathogenic bacteria

## Abstract

**Background:**

Many medicinal plant species from the Araripe Basin are widely known and used in folk medicine and for commercial manufacturing of phytotherapeutic products. Few ethnobotanical and pharmacological studies have been undertaken in this region, however, in spite of the great cultural and biological diversity found there.

**Materials and Methods:**

Extracts of 11 plant species collected from Ceará state, Brazil, were subjected to the brine shrimp lethality test in order to detect potential sources of novel cytotoxic, antitumor compounds. The larvicidal activity, based on the percentage of larval mortality, was evaluated after 24 h exposure to the treatments.

**Results:**

All species tested showed good larvicidal activity as compared to a reference compound and literature data. The extract from **Vanillosmopsis arborea** was the most active with an LC_50_ of 3.9 *μ*g/ml. Best results were shown by **Lantana montevidensis** against **Pseudomonas aeruginosa** [minimum inhibitory concentration (MIC) 8*μ*g/ml] and **Escherichia coli** (MIC 32 *μ*g/ml), *Zanthoxylum rhoifolium* against E. coli (MIC, 256 *μ*g/ml) and **Staphylococcus aureus** (MIC 64 *μ*g/ml) and **Croton zenhtneri** against *S. aureus* (MIC 64 *μ*g/ml).

**Conclusion:**

Chemical tests indicated that a wide variety of natural product classes was present in those extracts that showed significant activities in the bioassays.

## INTRODUCTION

Although progress has been made through conventional chemistry and pharmacology in producing effective drugs, the plant kingdom may provide useful sources of new anti-ulcer compounds for development as pharmaceutical entities or, alternatively, as simple dietary adjuncts to existing therapies.[1] The use of natural resources by rural communities in northeastern Brazil has been examined in greater depth. These communities practice subsistence agriculture, cattle ranching, and harvest both woody and non-woody forest products, sometimes associated with agroforest systems.[2]

Despite a less diversified flora, when compared with other regions of Brazil, Ceará presents numerous species widely used in popular medicine. The cytotoxicity and antitumoral effects, such as those produced by antifungal activity of extracts from Ceará’s flora, have been reported.[3]

Many medicinal plant species from the Araripe Basin are widely known and used in folk medicine and for commercial manufacturing of phytotherapeutic products. Few ethnobotanical and pharmacological studies have been undertaken in this region, however, in spite of the great cultural and biological diversity found there. In an effort to discover new lead compounds, many research groups screen plant extracts to detect secondary metabolites with relevant biological activities.

In this regard, several simple bioassays have been developed for screening purposes,[4] and some of them were used in this screening. Thus, *Artemia salina* larvae have been used as a target organism to detect bioactive compounds in plant extracts and toxicity to this crustacean has a good correlation with anti-tumor[5] and anti-*Trypanosoma cruzi*[6] activities. More recently, it has been shown that there is a very good correlation between the median lethal concentrations (LC_50_) of plant extracts to brine shrimp larvae and the median lethal doses (LD_50_) of the same extracts, administered orally in mice.[7]

As part of our program to evaluate medicinal plants from northeastern Brazilian flora, the aim of this study was to screen medicinal plant extracts that could be useful for the development of new tools for the control of infectious diseases. While pursuing this goal, we initiated a systematic evaluation of extracts from the Araripe Basin plant species, the brine shrimp (*A. salina* Leach) lethality assay (BSLA).

## MATERIALS AND METHODS

### Plants and sample preparation

A total of 11 plant species were collected randomly from the medicinal plant garden of the Universidade Regional do Cariri and Floresta Nacional do Araripe, Brazil. The taxonomic identities of these plants were confirmed by a botanist and the voucher specimen numbers of the plants were deposited in the Herbário Caririense Dárdano de Andrade Lima. Different parts of the plants (leaves, barks and stem barks) were dried, powdered and exhaustively extracted with ethanol at room temperature for 2 days.

### Phytochemical prospection

Phytochemical tests to detect the presence of heterosides, saponins, tannins, flavonoids, steroids, triterpenes, coumarines, quinones, organic acids and alkaloids were performed following the method described by Matos.[8] These tests are based on visual observation of color modification or precipitate formation after addition of specific reagents.

### Brine shrimp cytotoxicity assay

*A. salina* encysted eggs were incubated in artigical seawater under light at 28°C. After incubation for 24 h, nauplii were collected with a Pasteur pipette and kept for an additional 24 h under the same conditions to reach the metanauplii stage. The samples (triplicate) to be assayed were dissolved in Tween 80 and diluted serially (1000, 250, 125, 100, 75 μg/ml) in seawater. Ten nauplii were added to each set of tubes containing the samples. Controls containing Tween 80 in seawater were included in each experiment. K_2_Cr_2_O_7_ was used as a positive control. Twenty-four hours later, the number of survivors was counted.

### Lethal concentration determination

The lethal concentrations of plant extracts resulting in 50% mortality of the brine shrimp (LC_50_) and 95% confidence intervals were determined from the 24-h counts and the dose–response data were transformed into a straight line by means of a trendline fit linear regression analysis; the LC_50_ was derived from the best-fit line obtained.[9]

### Antibacterial assays

The antibacterial activities of the extracts were investigated by employing a microdilution method, recommended by NCCLS M7-A6.[10] The assay was carried out with four bacterial species obtained from Fundação Oswaldo Cruz-FIOCRUZ: **Staphylococcus aureus** (ATCC 6538), **Pseudomonas aeruginosa** (ATCC 15442), *Klebsiella pneumoniae* (ATCC 10031) and **Escherichia coli** (ATCC 25922).

Brain Hear Infusion (BHI 3.8%) broth was used for bacterial growth (24 h, 35 ± 2°C). The inoculum was an overnight culture of each bacterial species in BHI broth diluted in the same media to a final concentration of approximately 1 × 10^8^ Colony Forming Unit - CFU/ml (0.5 nephelometric turbidity units – McFarland scale). After this, the suspension was diluted to 1 × 10^6^ CFU/ml in 10% BHI. Hundred milliliters of each dilution was distributed in 96-well plates plus extracts in different concentrations, achieving 5 × 10^5^ UFC/ml as the final concentration of the inoculum. Extracts were dissolved in distilled water and dimethyl sulfoxide (DMSO) to a concentration of 103 mg/ml. Further serial dilutions were performed by addition of BHI broth to reach a final concentration in the range of 512 - 8 mg/ml. All experiments were performed in triplicate, and the microdilution trays were incubated at 35 ± 2°C for 24 h. Antibacterial activity was detected using a colorimetric method by adding 25 ml of resauzurin, staining (0.01%) aqueous solution in each well at the end of the incubation period. The minimal inhibitory concentration (MIC) was defined as the lowest essential oil concentration able to inhibit the bacterial growth, as indicated by resauzurin staining (bacterial dead cells are not able to change the staining color when visually observed – blue to red).

## RESULTS AND DISCUSSION

Since demonstrating activity in a bioassay is the first necessary step in the drug development process from ethnomedical systems, some extracts from reputed medicinal plants were screened for *in vitro* toxicity using brine shrimp test and antibacterial activities, in order to search for biological activity. This method allows the use of smaller extract quantities and the testing of a larger number of samples and dilutions in a short time. The species studied were *Plectranthus amboinicus* (#3037), *Plectranthus barbatus* (#3038), *Zanthoxylum rhoifolium* (#38231), *Stryphnodendron rotundifolium* (#33621), *Lantana camara* (#1662), **Lantana montevidensis** (#1663), *Guapira graciliflora* (#4023), **Croton zenhtneri** (#1619), *Dimorphandra gardineriana* (#1527), **Vanillosmopsis arborea** (#43291), and *Piper tuberculatum* (#43042), as shown in Table 1.

It was also important to separate and identify the active constituents chemically. Table 1 presents a summary of the classes of compounds that we have shown by chemical tests to be present in the plants used in this survey. The LC_50_ values obtained after the analysis of extracts from medicinal plants from the northeast of Brazil are shown in Table 2. All plant extracts analyzed demonstrated toxicity to brine shrimp (LC_50_ < 1000 μg/ml). These results suggested the presence of bioactive plant compounds and required further examination using elaborate bioassays which would detect pharmacological proprieties of a more specific nature.

**Table 1:** Parts of the plant used in the preparation of extracts, amounts and yield and identification of the main chemical classes

Plant	Part used	Yield(%)	Chemical classes
*P. amboinicus*	Leaves	1.08	Tannins/flavonoids
*P. barbatus*	Leaves	2.56	Tannins/flavonoids
*Z. rhoifolium*	Leaves	3.40	Tannins/flavonoids
*S. rotundifolium*	Bark	7.00	Tannins/flavonoids/alkaloids
*L. camara*	Leaves	5.00	Tannins/flavonoids/alkaloids
*L. montevidensis*	Leaves	5.50	Tannins/flavonoids/alkaloids
*G. graciliflora*	Stem	7.90	Tannins/flavonoids
*C. zenhtneri*	Leaves	9.50	Tannins/flavonoids/alkaloids
*D. gardineriana*	Stem bark	17.70	Flavonoids
*V. arborea*	Stem	8.00	Tannins/flavonoids/alkaloids
*P. tuberculatum*	Leaves	2.80	Tannins/flavonoids/alkaloids

**Table 2:** Larvicidal activities of ethanol extracts of various plant species against the larvae of *A. salina*

Scientific name	CL_50_ (μg/ml)
*P. amboinicus*	8.2
*P. barbatus*	5.3
*Z. rhoifolium*	363.0
*S. rotundifolium*	270.0
*L. camara*	50.0
*L. montevidensis*	13.0
*G. graciliflora*	478.0
*C. zenhtneri*	562.0
*D. gardineriana*	199.0
*V. arborea*	3.9
*P. tuberculatum*	10.2

According to Meyer *et al*, who classified crude extracts and pure substances into toxic (LC_50_ value < 1000 μg/ml) and non-toxic (LC_50_ value > 1000 μg/ml), all extracts tested showed good brine shrimp larvicidal activity.

Several new antibacterial agents are currently being developed in response to the emergence of bacterial resistance to existing drug. New vegetal sources presenting with antimicrobial activity and low toxicity could be a viable alternative to poor communities inhabiting the areas where the species are found, due to their low cost and easy accessibility.[11] The antibacterial activity of ethanolic extract of various plants presented significant results against the pathogenic microorganisms tested.

In relation to the antibacterial assay, *L. montevidensis* was the most active, presenting lower MICs against *P. aeruginosa* (MIC = 8 μg/ml), one of the most clinically important microorganisms because of its high resistance potential to conventional antibiotics. The extract was effective against *E. coli* (MIC 32 μg/ml) too. Similar studies done with this species have demonstrated activity against standard pathogenic and multiresistant strains of *E. coli* (Ec 27) (16 μg/ml).[12]

*Z. rhoifolium* extract was effective against the gram-negative *E. coli* (MIC 256 μg/ml) and the gram-positive *S. aureus* (MIC 64 μg/mL). Previous studies indicated that the leaves and fruit essential oils were bioactive against *S. aureus*, *K. pneumoniae* and *Salmonella* sp.; however, the essential oil from the flowers was inactive.[13] In the antimicrobial study, it was verified that among the analyzed strains, *S. aureus*, *Salmonella choleraesuis* and *Shigella flexneri* were sensitive to the essential oil, with inhibition halos of 15, 13 and 10 mm, respectively.[14]

*C. zenhtneri* presented significant activity only against the gram-positive *S. aureus* (MIC 64 μg/ml). The essential oil from the leaves of this species demonstrated antibacterial activity against *E. coli, S. aureus, Streptococus β-haemolyticus*, but the best result was against *S. flexneri* (CIM 50 μg/ml.[15] It was evidenced that five species presented activity against only one strain, *P. barbatus* against *S. aureus* (CIM 128 μg/ml), *S. rotundifolium* against *E. coli* (CIM 128 μg/ml), *L. camara* against *E. coli* (CIM 256 μg/ml), *G. graciliflora* against *K pneumoniae* (CIM 512μg/ml), *D. gardineriana* against *S. aureus* (CIM 128 μg/ml).

*V. arborea* was sensitive against all strains only at the highest concentration, showing a low antibacterial activity, whereas *P. amboinicus* and *P. tuberculatum* were not active against the used strains as shown Table 3.

**Table 3:** Antibacterial activity of ethanol extracts of various plant species

Plant	Microorganisms MIC (μg/ml)			
	*S. aureus* (ATCC 6538)	*E. coli* (ATCC 25922)	*K. pneumoniae* (ATCC)	*P. aeruginosa* (ATCC 15442)
*P. amboinicus*	≥1024	≥1024	≥1024	≥1024
*P. barbatus*	128	≥1024	≥1024	≥1024
*Z. rhoifolium*	64	256	512	512
*S. rotundifolium*	>1024	128	512	>1024
*L. camara*	512	256	512	256
*L. montevidensis*	512	32	512	8
*G. graciliflora*	≥1024	≥1024	512	≥1024
*C. zenhtneri*	64	512	≥1024	≥1024
*D. gardineriana*	128	512	≥1024	512
*V. arborea*	512	512	512	512
*P. tuberculatum*	≥1024	≥1024	≥1024	≥1024

The most promising extracts for the activity of toxicity were the aqueous extracts of *V. arborea, P. barbatus* and *P. amboinicus*, which have been used in the traditional medicine as analgesic and anti-inflammatory agents. In addition, the extracts of *Z. rhoifolium, S. rotundifolium, L. camara, L. montevidensis, G. graciliflora, C. zenhtneri, D. gardineriana* and *P. tuberculatum* showed significant lethality to brine shrimp.

These extracts can be regarded as promising candidates for a plant-derived antitumor compound. In fact, the essential oil of *V. arborea* was found to present the highest larvicidal activity.[16] The activity results of species belonging to Piperaceae family were found to be consistent with existing phytochemical knowledge of these plants as a source of cytotoxic and antitumor compounds.[17] It is possible that a broad range of structurally diverse compounds contributes to the overall pharmacological activity of the extracts and synergistic effects between active principles may exist.

The antibacterial activity demonstrated in this study for some extracts are relevant, since the best results against gram-negative strains that are often more resistant than gram-positive. This fact may be correlated with the polarity of the polar chemicals (tannins, flavonoids and alkaloids) present in extracts that can interact with the chemical composition of the polar cell structure of this bacterial type.
